# *Candida* Colonization in the Respiratory Tract: What Is the Significance?

**DOI:** 10.3389/fmed.2020.598037

**Published:** 2021-02-04

**Authors:** Jiao Liu, Yue-Tian Yu, Chun-Hui Xu, De-Chang Chen

**Affiliations:** ^1^Department of Critical Care Medicine, Ruijin Hospital, Shanghai Jiao Tong University School of Medicine, Shanghai, China; ^2^Department of Critical Care Medicine, Ren Ji Hospital, Shanghai Jiao Tong University School of Medicine, Shanghai, China; ^3^Clinical Laboratory Center, Institute of Hematology and Blood Diseases Hospital, Chinese Academy of Medical Sciences and Peking Union Medical College, Tianjin, China

**Keywords:** *Candida*, colonization, ventilator associate pneumonia, critical ill patients, bioflim

## Introduction

*Candida* spp. is one of the most important components of human microecology. Among hospitalized patients, the isolation rate of *Candida* spp. by active screening is about 15%, while in critically ill patients, the rate can be as high as 25% (1). Although microbial colonization plays an important role in secondary infections, *Candida* pneumonia is seldom documented even in the intensive care unit (ICU). Thus, the common consensus is that anti-*Candida* therapy is rarely necessary in most cases and it should be considered as colonization in which *Candida* spp. are isolated from the respiratory tract (RT) (2).

The co-existence of bacteria and fungi has raised great concern in the last decade. It has been indicated by some studies that *Candida* colonization in the RT might be an independent risk factor that could promote ventilator-associated pneumonia (VAP) and even change the antibiotic resistance patterns of pathogenic bacteria by polymicrobial biofilm formation (3, 4). Therefore, the significance of *Candida* colonization in RT remains controversial, and many clinical problems need to be reinterpreted.

## Current Situation Regarding *Candida* RT Colonization and *Candida* Pneumonia in Critical ILL Patients

The rate of *Candida* spp. isolation in the RT is relatively high, especially in those with mechanical ventilation (MV) (3). However, whether VAP can be caused by *Candida* spp. remains controversial and the main reasons for this are listed as follows: (1) No matter what the pathogenic microorganism is, the diagnosis of VAP is still difficult due to the lack of pathological evidence. The clinical diagnostic criteria for suspected VAP are not specific, and it is difficult to distinguish between colonization and infection (5). (2) The understanding of the importance of bacterial and fungal co-existence is not deep enough. Some microbiological laboratories have not conducted further analysis when fast-growing *Candida* spp. are isolated from RT samples. What's more, only filamentous fungi isolation were reported in some institutions (6). (3) It is widely accepted that the cutoff value for the number of pathogenic bacteria for VAP diagnosis is 10^3^ cfu/mL (protected specimen brush sample) or 10^4^ cfu/mL (bronchoalveolar lavage fluid sample), but such a threshold has not yet been established for *Candida* (5). Therefore, *Candida* pneumonia must be diagnosed by histopathology.

Hence, it is generally thought that *Candida* pneumonia is quite rare in the ICU, and the guidelines for the management of *Candida* spp. of both the IDSA and ESCMID do not recommend antifungal treatment unless there is clear histological evidence of infection (2, 7).

Alveolar macrophages act as the first line of defense against *Candida* in critically ill patients. Toll-like receptor (TLR) induces a Th1 cytokine pattern to increase the levels of IFN-γ and TNF-α to facilitate the clearance of *Candida* spores from the alveoli. What is more, other researches have also indicated that IFN-γ favors the intracellular killing of the fungus after internalization in professional phagocytes (8). Thus, it can be inferred that *Candida* pneumonia may not exist in the ICU. An autopsy study with 135 patients who died of pneumonia showed that among them, 77 (57%) severely affected patients had *Candida* airway colonization during their hospital stay. However, none of these cases was pathologically confirmed as *Candida* pneumonia (9). Meanwhile, one controlled before-after study in a microbiology laboratory at Illinois University showed that limiting the identification of respiratory secretions (only filamentous fungi were reported) could reduce the prescription of antifungal drug treatment (21 vs. 39%) and shorten the length of hospital stay (10.1 vs. 12.1 days) compared with full identification (all rapidly growing yeasts were reported), *p* < 0.05 (6).

## Impact of Bacteria and Fungi Co-existence on Microorganism Colonization and Drug Resistance Patterns—Insight From *in vitro* and Animal Studies

What should ICU physicians do when they receive a microbial culture report which indicates that *Candida* spp. are growing fast in airway secretions? The practice guidelines recommend that antifungal therapy should not be routinely used in those with *Candida* airway colonization (2, 7). However, should *Candida* colonization in the airway of critically ill patients simply be ignored? Some *in vitro* experiments on the co-existence of bacteria and fungi came to different conclusions.

The cell wall of *Candida* spp. is combined with polysaccharides and proteins. Among them, Beta-glucan (BG) is a proinflammatory factor that can cause dysfunction of macrophages and neutrophils in alveoli as well as reduce the production of reactive oxygen species (10). It is also reported that there is a strong interaction among *Candida*, Gram-positive and Gram-negative bacteria through quorum sensing (QS) molecules, and the extensive interaction of metabolic processes and intercellular communication among them are the basis of synergistic and antagonistic interactions (11). Through an observational study of rats injected with active *Candida albicans*, it was found that the increased production of cellular inflammatory factors, including interleukin-6, interferon-γ and tumor necrosis factor-α, inhibited phagocytosis by alveolar macrophages. This phenomenon led to changes in airway microecology, and an increase in the airway colonization rate of *Pseudomonas aeruginosa* was found (12). Moreover, this effect was not unique to *Pseudomonas aeruginosa*. Another study showed that *Candida* colonization was also beneficial for the colonization of *Staphylococcus aureus* and *Enterobacteriaceae*, which led to an increase in bacterial pneumonia (13).

**Figure 1:**
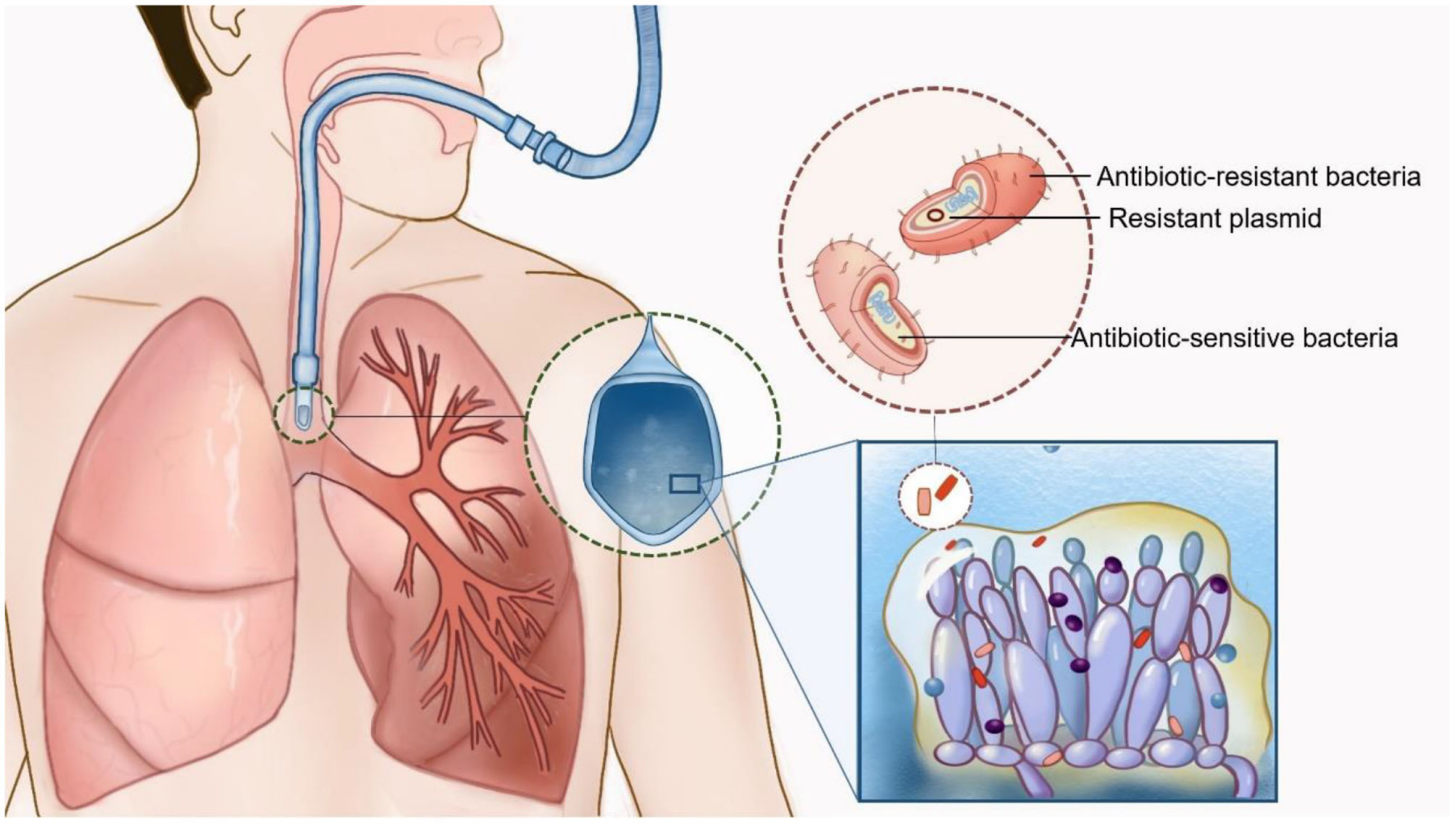
Interaction of *Candida* spp. and bacteria in patients with mechanical ventilation. *Candida* biofilms are easily found on the respiratory tract or the surfaces of endotracheal tubes. Biofilms not only have a protective effect on *Candida* but also have a strong adsorption effect on co-existing bacteria. Multidrug-resistant bacteria could be isolated by the transmission of drug-resistant plasmid transmission and polymicrobial biofilm formation (Drawn by Chunhui Xu).

*Candida* biofilms show a reticular structure composed of *Candida* spores and hyphae and are easily found on the surfaces of artificial materials (such as endotracheal tubes). The biofilm matrix contains polysaccharides, proteins and other unknown components, which show strong adhesion and are difficult to remove (14) (Figure 1). Biofilms not only have a protective effect on *Candida* but also have a strong adsorption effect on co-existing bacteria. Animal experiments and electron microscopic studies show that bacteria and fungi can produce small molecules to interact with each other and change their morphology, function and growth environment, resulting in bacteria that are firmly adsorbed between *Candida* spores or biofilms. Such structures are difficult to remove. Even though the spore activity of some *Candida* spp. is decreased, the adsorption phenomenon is still observed (4, 15).

*Candida* colonization can also change the virulence and/or host immune function of colonized bacteria. A series of animal experiments have shown that after the mixed inoculation of *Candida* and bacteria in the airway of mice, even if the number of inoculated *Candida* is very small, the bacterial load still occupies a high percentage of the alveoli. It has been suggested that the presence of *Candida albicans* protects the bacteria from clearance by normal alveolar macrophages (16). *Acinetobacter baumannii* can affect the morphology of *Candida albicans* through the QS molecule N-acyl homoserine lactone, whereas farnesol is the main QS molecule of *Candida albicans* (11). This can affect the movement ability and virulence factor expression of *Acinetobacter baumannii*. An animal experiment has also found that the degree of alveolar invasiveness of *Acinetobacter baumannii* in mice with *Candida* colonization during pneumonia is much higher than that of *Acinetobacter baumannii* during pulmonary infection (17).

The existence of biofilms can also increase the resistance of bacteria to antibiotics. It is showed that *Staphylococcus aureus* could form a single biofilm (monoculture biofilm) in serum, but its integrity was poor, and it was easy to dissociate. If there is co-growth with *Candida albicans, Staphylococcus aureus* can form microcolonies on the fungal biofilm, which is closely connected to the bottom hyphae “scaffold,” to form a multi-bacterial biofilm (polymicrobial biofilm) (Supplementary Figure 1). *Staphylococcus aureus* matrix staining showed different phenotypes of multi-bacterial biofilms and single cell membranes (18), indicating that *Staphylococcus aureus* may be encapsulated in the matrix secreted by *Candida albicans*, resulting in an increase in its resistance to vancomycin.

Further studies showed that in the environment of multi-bacterial biofilm formation, 27 *Staphylococcus aureus*-specific proteins were identified by gel electrophoresis, some of which could upregulate the expression of L-lactate dehydrogenase I, confer the ability to resist host-derived oxidative stress to bacteria and enhance resistance to antibiotics, while other proteins could downregulate the expression of the virulence factor CodY (19). These findings suggest that the occurrence of VAP caused by MRSA in patients with *Candida albicans* airway colonization is not only the result of the expression of QS molecules but can also be attributed to the differential regulation of specific drug resistance genes and virulence factors. Similar results have been obtained in other studies of Gram-negative bacteria (20, 21). *In vitro* studies suggest that there is mutual induction of the process of the co-existence of bacteria and fungi, so it is necessary to further describe and study the complex interactions between pathogens at the molecular level. The transition from basic research to clinical research may help to design new treatment or prevention and control strategies for bacterial and fungal superinfection.

## Impact of Bacteria and Fungi Co-existence on Microorganism Colonization and Drug Resistance Patterns—Insight From A Clinical Study

Clinical studies have pointed out that the isolation rate of *Candida* from the RT of ICU patients with MV could be as high as 50%, which prolonged the median hospital stay (59.9 vs. 38.6 days, *p* = 0.006) or even increased the hospital mortality (34.2 vs. 21.0%, *p* = 0.003) (22). Moreover, it might be associated with persistent immunosuppression and inflammation (23). *Candida* airway colonization and its concomitant secretion of inflammatory factors may affect host cellular immune function, especially in immunosuppressed hosts with severe monocyte and lymphocyte dysfunction, which results in a decrease in the effective clearance of bacteria and fungi and an increase in the incidence of VAP (24).

However, the effect of *Candida* RT colonization on bacterial colonization and antibacterial resistance patterns has always been controversial in clinical research. It is still unclear whether *Candida* airway colonization could increase the incidence of VAP and whether patients with *Candida* airway colonization can benefit from antifungal therapy (Supplementary Table 1).

One early prospective cohort study reported that *Candida* RT colonization could increase the incidence of VAP caused by *Pseudomonas aeruginosa* (9 vs. 4.8%, *p* = 0.048), and *Candida* RT colonization was proven to be an independent risk factor (18). Similarly, another single-center retrospective case-control study indicated that antifungal therapy in those with *Candida albicans* airway colonization could prevent the occurrence of *Pseudomonas aeruginosa* VAP (25). Some studies have also pointed out that *Candida* airway colonization is associated with the pathogenesis of *Acinetobacter baumannii* VAP. In addition, another cohort study showed that aerosol inhalation of amphotericin B in patients with MV significantly reduced the *Candida* load in the airway but did not change the morbidity due to VAP or mortality during the ICU stay (26, 27).

The EMPIRICUS study is a randomized trial to evaluate the efficacy of micafungin for the treatment of patients with *Candida* colonization in multiple sites and sepsis with organ failure (28). The study noted that the incidence of VAP and the 28-days mortality during the ICU stay did not decrease in the micafungin group compared with those in the placebo group (32 vs. 39.8%, *p* > 0.05). Therefore, the above studies led to a change in the understanding of the co-existence of bacteria and fungi and their effects on immune function in clinical studies. FUNGIBACT, as a prospective cohort study, included 146 patients with MV for more than 96 h. After adjusting for the immune index mHLA-DR, it was concluded that there was no correlation between airway *Candida* colonization and the incidence of VAP [HR: 0.98; 95% CI (0.59–1.65), *p* = 0.95] (29). Another retrospective study reviewed 269 systemic lupus erythematosus patients with hospital-acquired pneumonia. Among them, 186 (69.1%) were found to have airway *Candida* colonization. Compared with that in the non-colonized group, the detection rate of multidrug-resistant bacteria was higher (58.6 vs. 36.1%, *p* < 0.001), and the secreted IgA and IL-17 levels returned to normal range faster after anti-fungal treatment, but this had no effect on 28-days mortality (14.5 vs. 10.8, *p* > 0.05) (30).

One meta-analysis about the influence of Candida spp. airway colonization on clinical outcomes in patients with VAP included four prospective studies, three retrospective studies, and one cross-sectional study (31). It revealed that those with airway *Candida* colonization had longer durations of MV. The most noteworthy feature of the meta-analysis is that patients with *Candida* colonization had higher 28-days mortality (RR: 1.64; 95% CI: 1.27–2.12) and ICU mortality (RR: 1.57; 95% CI: 1.26–1.94) than those without *Candida* colonization. Although it has included almost all the clinical research about airway *Candida* colonization with high quality, limitations still exist. First, attributable mortality rate could hardly find in these studies duo to the effects of confounding factors and the insufficient sample size. Second, a highly heterogeneity could be recognized in the baseline of the enrolled patients.

Reasons for MV, severity of VAP, antibiotic exposures before the diagnosis of VAP and the immune state was probably diverse among studies.

## Perspective

Although “*Candida* pneumonia” is rarely confirmed in critically ill patients, *Candida* airway colonization may affect bacterial colonization and antibacterial resistance patterns, playing an important role in the development of bacterial pneumonia. However, the conclusions of current clinical studies are not consistent. Future clinical studies are needed to re-evaluate the potential benefits of pre-emptive antifungal therapy for preventing VAP.

## Author Contributions

Y-TY and JL: conception and design. D-CC: administrative support. C-HX: provision of study materials or patients. Y-TY and C-HX: data analysis and interpretation. All authors: collection and assembly of data, manuscript writing, and final approval of manuscript.

## Conflict of Interest

The authors declare that the research was conducted in the absence of any commercial or financial relationships that could be construed as a potential conflict of interest.
