# Supplementary material for: Candida Colonization in the Respiratory Tract: What Is the Significance?
Source: Front Med (Lausanne). 2021 Feb 4;7:598037. doi: 10.3389/fmed.2020.598037 (PMC7889970; doi:10.3389/fmed.2020.598037)
Supplement: Supplementary file 1 [file Data_Sheet_1.PDF]

Table 1 Researches focused on the impact of *Candida spp.* airway colonization

Author	Study design	Population	Number of patients included	Main results of *Candida spp.* airway colonization
Marie-Soleil Delisle et al, 2008[22]	Retrospective cohort study	Clinical suspicion of VAP	639	Increased hospital stay and hospital mortality
Marie-Soleil Delisle et al, 2011[24]	Retrospective cohort study	Clinical suspicion of VAP	274	Increased hospital stay and hospital mortality
Maël Hamet et al, 2012[3]	Prospective observational study	Suspected VAP	323	Increased hospital mortality and MDR pathogens isloation
Martin Albert et al, 2014[21]	Randomized controlled trial	Clinical suspicion of VAP	89	Associated with persistent inflammation and immunosuppression
Farzin Khorvash et al, 2014[27]	Cross-sectional study	Clinical suspicion of VAP	38	Not associated with mortality
Jean-Francois Timsit et al, 2016[28]	Double-blind placebo-controlled study	ICU acquired sepsis and multiple Candida colonization	260	Empirical treatment with micafungin did not increase fungal infection-free survival at day 28.
Yi Huang et al, 2018[23]	Observational study	Clinical suspicion of VAP	194	Increased hospital mortality and MDR pathogens isolation
Jean-Francois Timsit et al, 2019[29]	Observational study	Patients receiving MV and presenting multiple organ failure	213	Not associated with VAP
Yuetian Yu et al, 2019[30]	Retrospective cohort study	SLE patients with HABP	269	Not associated with mortality
Dong Huang et al, 2020[31]	Meta-analysis	Clinical suspicion of VAP	1661	Associated with longer durations of MV and higher mortality

ICU, Intensive Care Unit; VAP, Ventilator Associated Pneumonia; SLE, Systemic Lupus Erythematosus; HABP, Hospital-Acquired Bacterial Pneumonia; MDR, Multiple Drug Resistance; MV, Mechanical Ventilation
