# Supplementary figures and images for: Candida Colonization in the Respiratory Tract: What Is the Significance?
Source: Front Med (Lausanne). 2021 Feb 4;7:598037. doi: 10.3389/fmed.2020.598037 (PMC7889970; doi:10.3389/fmed.2020.598037)

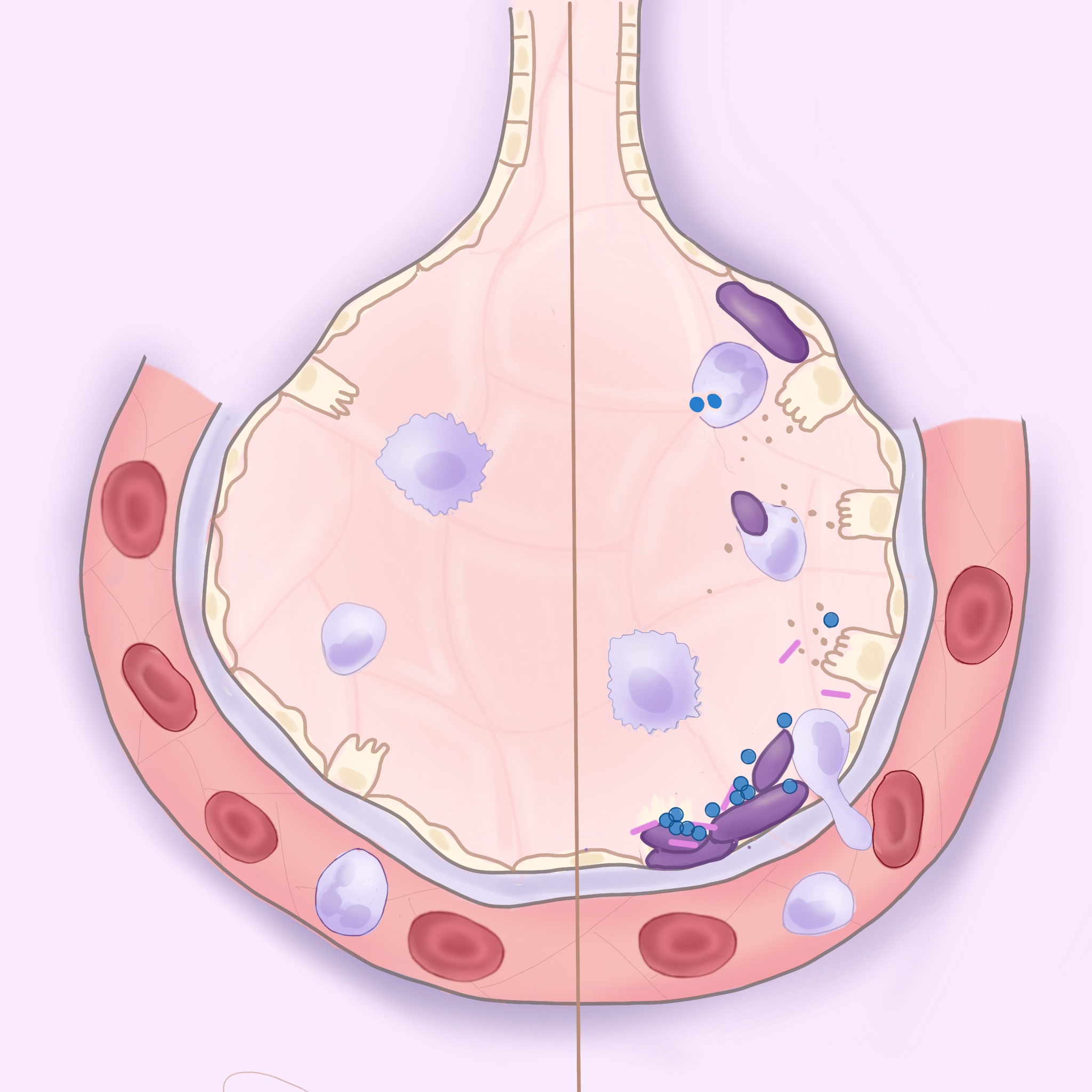

Supplement: Supplementary file 2 [file Image_1.JPEG]
